# Thermal Stability and Sublimation Pressures of Some Ruthenocene Compounds

**DOI:** 10.3390/ma3021172

**Published:** 2010-02-15

**Authors:** M. Aslam Siddiqi, Rehan A. Siddiqui, Burak Atakan, Nina Roth, Heinrich Lang

**Affiliations:** 1 Thermodynamics, IVG, Faculty of Engineering, and CeNIDE, University of Duisburg Essen, Lotharstr. 1, 47057 Duisburg, Germany; E-Mails: rehan.siddiqui@uni-due.de (R.A.S.); burak.atakan@uni-due.de (B.A.); 2 Department of Inorganic Chemistry, Institute of Chemistry, Faculty of Science, Chemnitz Technical University, Straße der Nationen 62, 09111 Chemnitz, Germany; E-Mail: nina.roth@chemie.tu-chemnitz.de (N.R.)

**Keywords:** ruthenocene, sublimation / vapor pressure, thermal stability

## Abstract

We set out to study the use of a series of ruthenocenes as possible and promising sources for ruthenium and/or ruthenium oxide film formation.The thermal stability of a series of ruthenocenes, including (*η*^5^-C_5_H_4_R)(*η*^5^-C_5_H_4_R´)Ru (**1)**, R = R´ = H (**3)**, R = H, R´ = CH_2_NMe_2_ (**5)**, R = H, R´= C(O)Me (**6)**, R = R´ = C(O)Me (**7)**, R = H, R´ = C(O)(CH_2_)_3_CO_2_H (**8)**, R = H, R´ = C(O)(CH_2_)_2_CO_2_H (**9)**, R = H, R´ = C(O)(CH_2_)_3_CO_2_Me (**10)**, R = H, R´= C(O)(CH_2_)_2_CO_2_Me (**11),** R = R´ = SiMe_3_), (*η*^5^-C_4_H_3_O-2,4-Me_2_)_2_Ru (**2)**, and (*η*^5^-C_5_H_5_-2,4-Me_2_)_2_Ru (**4)** was studied by thermogravimetry. From these studies, it could be concluded that **1**–**4, 6** and **9–11** are the most thermally stable molecules. The sublimation pressure of these sandwich compounds was measured using a Knudsen cell. Among these, the compound **11** shows the highest vapor pressure.

## 1. Introduction

**Figure 1:**
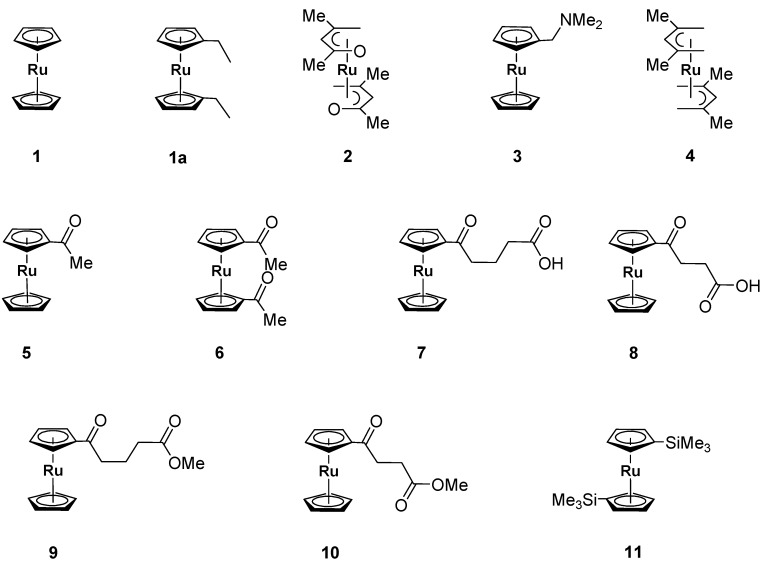
Ruthenocenes **1–11**.

Ruthenium thin films found many applications, for example, as a catalyst in ammonia synthesis, and in the production of fine chemicals [1]. In addition, they are of importance in the manufacture of bottom electrodes in the filed of microelectronics [2]. It was shown that several ruthenium-based sandwich compounds like ruthenocene ([(*η*^5^‑C_5_H_5_)_2_Ru]) [3,4,5,6], bis(ethylcyclopentadienyl) ruthenium ([(*η*^5^‑C_5_H_4_Et)_2_Ru]); **1a** [7,8,9,10], tris(2,4-pentanedionato)ruthenium(III) ([(C_5_H_7_O_2_)_3_Ru]) [11], and tris(2,2,6,6-tetramethyl-3,5-heptanedionato)ruthenium(III) ([(C_11_H_19_O_2_)_3_Ru]) [11,12] can be successfully used as precursor molecules for the deposition of ruthenium and/or ruthenium oxide layers applying either CVD (= chemical vapor deposition) and ALD (= atomic layer deposition) processes (when volatile), or dip- and spin-coating techniques (when non-volatile) [13]. One disadvantage associated with this family of compounds is that depending on the nature of the cyclopentadienyl-bonded organics groups, the respective films may contain (some) carbon impurities [13]. In this respect, we set out to study the use of ruthenocenes **1–11** (Figure 1) as possible and promising sources for ruthenium and/or ruthenium oxide film formation by the techniques described earlier [13]. Here we report their vapor and sublimation pressure determinations. 

While for ruthenocene **1** some vapor pressure data are available in the temperature range of 75 to 93 °C [14,15], the appropriate data for **4** were determined at 82 °C [16]. Nevertheless, for **2**, **3**, and **5–11**, the corresponding data are completely missing. This prompted us to systematically investigate their thermal stabilities and vapor pressures.

## 2. Results and Discussion

**Figure 2:**
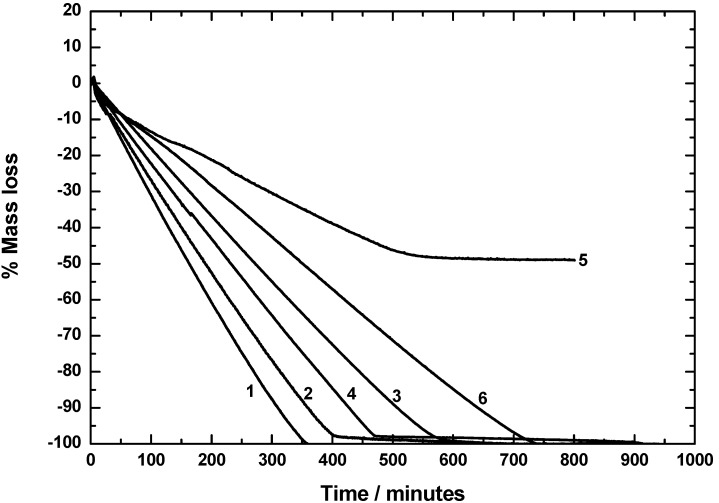
TG curves in the isothermal mode for **1** and **3**–**5** at 95 °C, **2** at 115 °C, and **6** at 144 °C.

**Figure 3:**
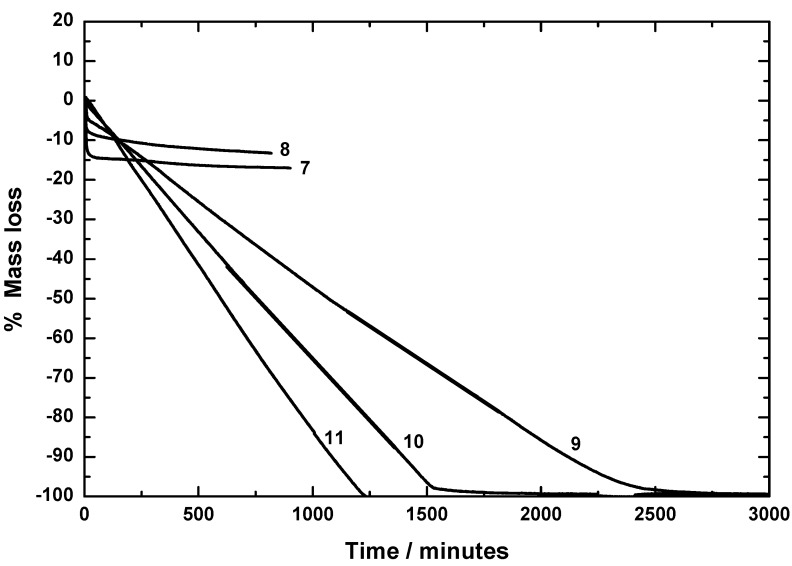
TG curves in the isothermal mode for **7–10** at 143 °C, and **11** at 90 °C.

The stability of ruthenocenes **1–11** (Figure 1) was investigated by applying isothermal thermogravimetry using DTA/TG equipment (Bähr STA 503). The experimental conditions were selected to simulate typical evaporator conditions (90 °C to 145 °C), the pressure was atmospheric and a well-defined amount of inert gas (100 cm³/min) was flowing on the top of the crucibles. Depending on the substance, different temperatures were selected to obtain a TG curve in a reasonable time and with reasonable accuracy. Thus, at a given constant temperature the mass loss as well as the DTA signal was determined. It should be noted that this does not correspond to the normal TG mode, where the temperature is changing with time. This is normally not the case in typical evaporators. A molecule can be considered as a good CVD precursor, when in isothermal TG studies a nearly linear mass loss as a function of time is found and no residual is left at the end. The theory of this process was established earlier and is reported in reference [18]. Typical results obtained from our TG measurements with ruthenocenes **1–11** are summarized and depicted in Figure 2 and Figure 3.

Ruthenocenes **1–4**, **6** and **9–11** evaporate completely without leaving any residue or less than 2%, indicating that the evaporation is taking place without any significant decomposition of the appropriate ruthenocene molecules (Figure 2 and Figure 3), and show a mass loss curve as expected by theory derived earlier [18]. The temperature for the isothermal experiments was 95 °C for **1**, **3** and **4** and 115 °C for **2**. 

Rethonecenes **5**, **7** and **8**, however, are not stable in the observed process window as can be clearly seen by the formation of a residue in isothermal TG measurements (Figure 2 and Figure 3). In the absence of any reductants, a thermolytic mechanism according to the decomposition of, for example, ferrocenes [19,20,21], titanocenes, ziconocenes, and hafnocenes [22] is most likely. Here, residues are formed composing of the appropriate metals along with metal carbides, carbon and/or oxygen containing impurities. 

For ruthenocenes **1**–**4**, **6** and **9**–**11**, which were found to be thermally stable (*vide supra*), sublimation pressure measurements were carried out at various temperatures under vacuum using the Knudsen effusion method as described previously in references [18,23]. This method is based on the kinetic theory of gases. As soon as the mean free path length is larger than the typical dimensions of an aperture (area: *A*) that separates the sublimating substance from its surroundings, the mass loss rate ($\frac{\Delta m}{\Delta t}$) is determined by the area of the orifice and the vapour pressure; this rate is the rate of effusion from the cell. The vapour/sublimation pressures *p* were determined from the measurement of the mass of the substance Δ*m* evaporated in a definite time Δ*t* in the Knudsen cell from equation

(1) $$p=\frac{\Delta m}{\text{K}\cdot A\cdot \Delta t}\cdot \sqrt{\frac{2\pi \cdot \text{R}\cdot T}{M}}$$

where *A* is the area of the aperture; R is the universal gas constant; *T* is the temperature in Kelvin; and *M* is the molar mass of the substance. The Clausing factor *K* of the aperture was calculated [24] using the relation

(2) $$\text{K}=1-0.5\left(\frac{l}{r}\right)+0.2{\left(\frac{l}{r}\right)}^{2}$$

where l is the thickness of the foil and r is the radius of the aperture.

The sublimation pressure resulting as a function of temperature was fitted to an equation and the coefficients A and B were determined for the best fit. The coefficients are given in Table 1.

(3) $$\log_{10}(p/\text{k Pa})=A_{i}-B_{i}/\;\left(T/K\right)$$

The enthalpy of sublimation for each compound was derived from these vapour pressure values (from the slopes of the log *p* *vs.* $\frac{1}{T}$ plots) and is also reported in Table 1.

**Figure 4:**
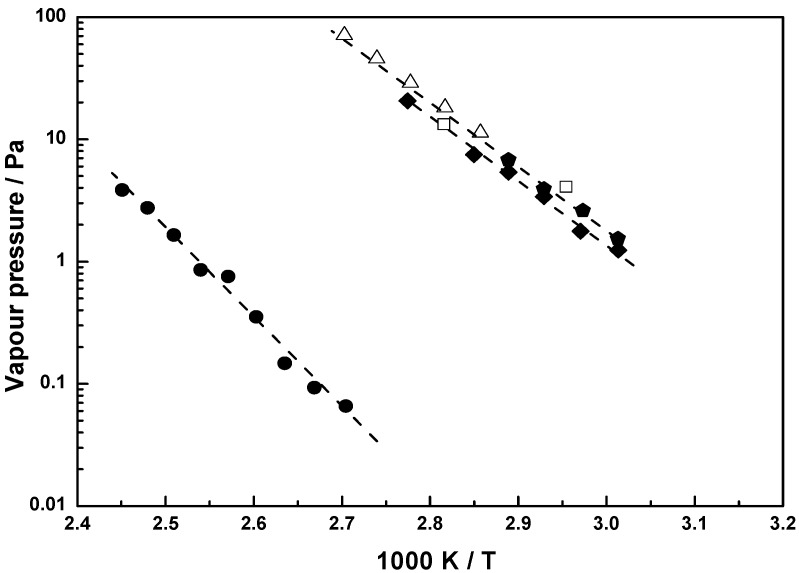
Vapor pressure for **1**: this work (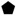), Cordes and Schreiner [14] (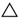); **4**: this work (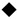), Kawano *et al.* [16,17] (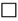); **6**: this work (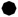); equation 3 (----).

For **1**, the sublimation pressure was measured in the temperature range 58 to 78 °C. The measured values and those calculated from equation (3) for **1** are plotted in Figure 4 as a function of $\frac{1}{T}$. The literature values of Cordes and Schreiner [14] for the sublimation pressure are also shown in Figure 4.

It can be seen that the vapor pressure values of **1** from reference [14] agree very well with the values obtained by the extrapolation of our studies to lower temperatures. Thus, our study extends the temperature range of available data for **1**. The enthalpy of sublimation for **1** is 100.52 ± 0.2 kJ mol^-1^ and is in good agreement with 98.78 kJ mol^-1^ found by Cordes and Schreiner [14] for the temperature range 83 to 97 °C. The sublimation pressure value for **1** given elsewhere [15] is 77.61 ± 1.5 kJ mol^-1^ (120 to 206 °C) differs from the values given above. 

The previously reported values for the vapor pressure of **4** (13.33 Pa at 82 °C and 4.1 Pa at 65 °C [16,17]) are also shown in Figure 4 together with our measured values using Knudsen effusion method.

**Figure 5:**
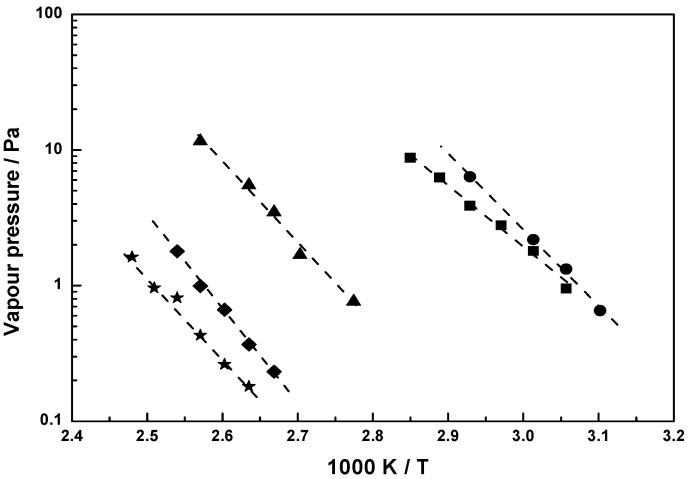
Measured vapor pressure for ruthenocenes **11** (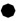), **3** (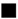), **2** (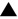), **9** (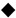), **10** (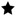); equation 3 (----).

Figure 5 shows the vapor pressures for **2**, **3** and **9–11** as a function of $\frac{1}{T}$. For these compounds, this is the first time that the vapor pressures have been reported. 

**Table 1:** The constants for equation (3) and the molar enthalpy of sublimation of compounds **1**–**4**, **6**, and **9**–**11**.

Compound	A_i_	B_i_	Δ*H_sub_*(exp) (*T*/K) / kJ ∙ mol^-1^
[(*η*^5^‑C_5_H_5_)_2_Ru] (**1**)	13.0	5249.99	100.52 (331–346) 98.78^a^ (356–370)
[(*η*^5^‑OC_6_H_9_)_2_Ru] (**2**)	13.45	5974.61	114.39 (360–384)
[(*η*^5^‑C_5_H_5_)(*η*^5^‑C_5_H_4_CH_2_NMe_2_)Ru] (**3**)	10.80	4505.81	86.27 (327–351)
[(*η*^5^‑C_7_H_11_)_2_Ru] (**4**)	12.54	5133.29	98.28 (331–360)
[(*η*^5^‑C_5_H_4_COCH_3_)_2_Ru] (**6**)	15.56	7310.25	139.97 (369–410)
[(*η*^5^‑C_5_H_5_)(*η* ^5^‑C_5_H_4_CO(CH_2_)_3_COOMe)Ru] (**9**)	11.98	5974.61	114,39 (379–403)
[(*η*^5^‑C_5_H_5_)(*η*^5^‑C_5_H_4_CO(CH_2_)_2_COOMe)Ru] (**10**)	14.87	6938.75	132.85 (374–394)
[(*η*^5^‑C_5_H_4_SiMe_3_)_2_Ru] (**11**)	14.24	5609.41	151.51 (331–346)

(a) Reference [14].

## 3. Experimental Section

**1** [25], **2** [26], **3** [27], **4** [28], **5** [29], **6** [29] and **7** [30] were prepared according to references. The other compounds were prepared according to the procedures given below. 

Compounds **8**, **9** and **10** were prepared according to the reaction sequence (4) starting from ruthenocene. Compound **11** [31] was synthesized by the consecutive reaction sequence (5).

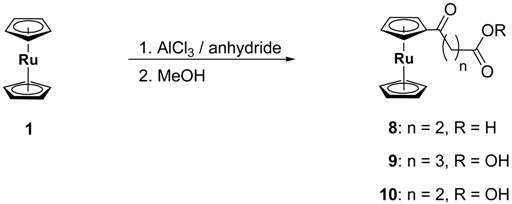
(4)

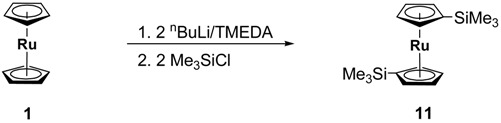
(5)


After appropriate work-ups, the sandwich compounds **9**, **10** and **11** could be isolated in the form of yellow solids in 14%, 23% and 73% yields (Experimental).

### 3.1. General Methods

All reactions were carried out under an atmosphere of purified nitrogen (O_2_ traces: copper oxide catalyst, BASF AG, Ludwigshafen; H_2_O: molecular sieve 4 Å, Aldrich Company) using standard Schlenk techniques. *n*-Hexane was purified by distillation from calcium hydride. Infrared spectra were recorded with a Nicolet FT-IR 200 spectrometer. ^1^H NMR spectra were recorded with a Bruker Avance 250 spectrometer operating at 250.130 MHz in the Fourier transform mode; ^13^C{^1^H} NMR spectra were recorded at 62.895 MHz. Chemical shifts are reported in *δ* units (parts per million) downfield from tetramethylsilane (*δ* = 0.0 ppm) with the solvent as the reference signal (^1^H NMR, CDCl_3_ *δ* = 7.26; ^13^C{^1^H} NMR, CDCl_3_ *δ* = 77.55). Melting points were determined using analytically pure samples, sealed off in nitrogen-purged capillaries on a Gallenkamp (typ MFB 595 010 M) melting point apparatus. Elemental analysis for **11** was performed with a Flashea, Thermo Electron Corporation analyser. 

***[(η^5^‑C_5_H_5_)-(η^5^‑C_5_H_4_CO(CH_2_)_2_COOH)Ru]* (8)**: To a suspension of aluminum trichloride (1.15 g, 8.65 mmol) in 100 mL of CH_2_Cl_2_ a mixture of [(*η*^5^‑C_5_H_5_)_2_Ru] (**1**) (2.00 g, 8.65 mmol) and succinic anhydride (0.87 g, 8.65 mmol) in 150 mL of CH_2_Cl_2_ was dropped slowly. The color changed from light yellow to orange-red. After 1 h refluxing the reaction was hydrolyzed. Insoluble products were removed by filtration through a pad of celite. The water phase was detached. The organic phase was washed with water (3 × 75 mL). The collected water phases were washed with 100 mL of CH_2_Cl_2_. The united organic phases were dried over MgSO_4_ and all volatile components were removed in membrane pump vacuum. Not reacted **1** (1.09 g, 4.71 mmol, 55%) could be isolated from the residue by column chromatography (aluminum oxide, 4 × 30 cm) and *n*‑hexane as a solvent. **8** was obtained with a mixture of ethanol/glacial acetic acid (7:3). Removing all the volatile compounds gave **8** as yellow crystals (0.19 g, 0.57 mmol, 7% based on **1**). 

C_14_H_14_O_3_Ru (331.33): Calc.: C, 50.75; H, 4.26%. Found: C, 50.55; H, 4.16%; mp: 198 °C; ^1^H-NMR (C_4_D_8_O): δ_H_ 5.11 (pt, *J* = 1.8 Hz, 2 H, C_5_*H*_4_), 4.74 (pt, *J* = 1.8 Hz, 2 H, C_5_*H*_4_), 4.61 (s, 5 H, C_5_*H*_5_), 2.90 (t, *J* = 6.6 Hz, 2 H, C*H*_2_-C(O)), 2.51 (t, *J* = 6.6 Hz, 2 H, C*H*_2_-CO_2_H); ^13^C{^1^H}-NMR (C_4_D_8_O): δ_C_ 197.9, 173.1, 84.0, 72.6, 71.6, 70.2, 33.2, 26.8; IR ***ῦ*** /cm (NaCl): 3435, 2963, 2962, 2855, 1710, 1660, 1454, 1428, 1404, 1384, 1344, 1261, 1211, 1172, 1101, 1077, 1032, 803, 628, 520, 508, 448, 432, 419.

***[(η^5^‑C_5_H_5_)-(η^5^‑C_5_H_4_CO(CH_2_)_3_COOMe)Ru]* (9)**: The reaction followed the scheme described for **8**. Aluminum trichloride (1.33 g, 10.00 mmol) in 100 mL of CH_2_Cl_2_ was reacted with [(*η*^5^‑C_5_H_5_)_2_Ru] (**1**) (2.30 g, 10.00 mmol) and glutaric anhydride (1.13 g, 10.00 mmol) in 100 mL of CH_2_Cl_2_. After refluxing the mixture for 3 h, the reaction was finished by addition of 150 mL of dry methanol followed by the removal of all volatile substances by membrane pump vacuum. Non-reacted **1** (1.45 g, 6.27 mmol, 63%) could be isolated from the residue by column chromatography (aluminum oxide, 4 × 30 cm) and *n*‑hexane as a solvent. **9** could be obtained with *n*-hexane/CH_2_Cl_2_ (1:1) as eluent. The volatile compounds were removed by membrane-pump vacuum leaving **9** as yellow solid material (0.5 g, 1.39 mmol, 14% based on **1**).

C_16_H_18_O_3_Ru (359.38): Calc.: C, 53.47; H, 5.05%. Found: C, 53.67; H, 5.08%; mp: 57 °C; ^1^H-NMR (CDCl_3_): δ_H_ 5.10 (pt, *J* = 1.8 Hz, 2 H, C_5_*H*_4_), 4.76 (pt, *J* = 1.8 Hz, 2 H, C_5_*H*_4_), 4.57 (s, 5 H, C_5_*H*_5_), 3.69 (s, 3 H, OC*H*_3_), 2.67 (t, *J* = 7.2 Hz, 2 H, C*H*_2_-C(O)), 2.39 (t, *J* = 7.2 Hz, 2 H, C*H*_2_-CO_2_CH_3_), 1.98 (q, *J* = 7.2 Hz, 2 H, C-C*H*_2_-C); ^13^C{^1^H}-NMR (CDCl_3_): δ_C_ 201.7, 173.9, 84.0, 73.7, 72.1, 70.8, 51.7, 37.8, 33.3, 20.1; IR ***ῦ*** /cm (NaCl): 3436, 3083, 2938, 1730, 1661, 1452, 1410, 1378, 1317, 1279, 1234, 1195, 1147, 1102, 1050, 1032, 1012, 994, 864, 819, 685, 515, 461. 

***[(η^5^‑C_5_H_5_)-(η^5^‑C_5_H_4_CO(CH_2_)_2_COOMe)Ru]* (10)**: The reaction followed the scheme described for **9**. Aluminum trichloride (1.39 g, 10.40 mmol) in 100 mL of CH_2_Cl_2_ was reacted with [(*η*^5^‑C_5_H_5_)_2_Ru] (**1**) (2.40 g, 10.40 mmol) and succinic anhydride (1.04 g, 10.40 mmol) in 150 mL of CH_2_Cl_2_. After refluxing the mixture for 3 h the reaction was finished by addition of 150 mL of dry methanol followed by the removal of all volatile substances by membrane pump vacuum. Non-reacted **1** (1.62 g, 7.00 mmol, 67%) could be isolated from the residue by column chromatography (aluminum oxide, 4 × 30 cm) and *n*‑hexane as a solvent. **9** could be obtained with *n*-hexane/CH_2_Cl_2_ (1:1) as eluent. The volatile compounds were removed in membrane-pump vacuum gave **10** as yellow solid material (0.81 g, 2.45 mmol, 23% based on **1**).

C_15_H_16_O_3_Ru (345.36): Calc.: C, 52.17; H, 4.47%. Found: C, 52.36; H, 4.67%; mp: 89 °C; ^1^H-NMR (C_4_D_8_O): δ_H_ 5.13 (pt, *J* = 1,8 Hz, 2 H, C_5_*H*_4_), 4.78 (pt, *J* = 1,8 Hz, 2 H, C_5_*H*_4_), 4.62 (s, 5 H, C_5_*H*_5_), 3.70 (s, 3 H, C*H*_3_), 2.98 (t, *J* = 6,7 Hz, 2 H, C*H*_2_-C(O)), 2.64 (t, *J* = 6,7 Hz, 2 H, C*H*_2_-CO_2_Me); ^13^C{^1^H}-NMR (C_4_D_8_O): δ_C_ 200.3, 173.7, 83.5, 73.8, 72.4, 70.8, 52.0, 33.7, 28.1; IR ***ῦ*** /cm (NaCl): 3434, 3091, 2996, 2951, 1727, 1665, 1456, 1437, 1414, 1401, 1366, 1257, 1214, 1162, 1102, 1077, 1048, 1026, 990, 955, 885, 846 820, 627, 582, 516, 445. 

***[(η^5^‑C_5_H_4_SiMe_3_)_2_Ru]* (11)**: To a suspension of [(*η*^5^‑C_5_H_5_)_2_Ru] (**1**) (1.70 g, 7.30 mmol) and tetramethylethylenediamine (2.73 mL, 18.25 mmol) in 100 mL of *n*-hexane, *n*-butyllithium (2.5 M, 7.30 mL, 18.25 mmol) was slowly added through a syringe at 25 °C. A yellow solid precipitated. After 16 h of stirring at this temperature the mixture was cooled to 0 °C and trimethylsilylchloride (2.33 mL, 1.98 g, 18.25 mmol) was added in a single portion, whereby a colorless solid precipitated. After stirring for 1 h at 0 °C the reaction mixture was hydrolyzed with 100 mL of water. The two phases were separated. The aqueous phase was washed with dichloromethane (3 × 50 mL). After combining the organic fractions and drying them over MgSO_4_ the solvent was evaporated in oil-pump vacuum. Chromatographic purification on a column (column size: 20 × 3 cm, aluminum oxide, *n*‑hexane) gave **1**. The second fraction gave the compound as yellow oil. After storing of the compound at 8 °C, yellow crystals were formed (2.02 g, 5.37 mmol, 73% based on **1**). 

C_16_H_26_RuSi_2_ (375.62): Calc.: C, 51.16; H, 6.98%. Found: C, 51.58 ; H, 7.22%; mp: 55 °C; ^1^H-NMR (CDCl_3_): δ_H_ 4.62 (pt, *J* = 1.6 Hz, 4 H, C_5_*H*_4_), 4.44 (pt, *J* = 1.6 Hz, 4 H, C_5_*H*_4_), 0.15 (s, 18 H, CH_3_); ^13^C{^1^H}-NMR (CDCl_3_): δ_C_ 75.4, 73.7, 71.0, 0.1; IR ***ῦ*** /cm (NaCl): 3102, 3092, 3081, 2953, 2895, 1634, 1440, 1419, 1402, 1379, 1366, 1303, 1246, 1184, 1159, 1101, 1080, 1053, 1032, 891, 865, 815, 751, 661, 629. 

### 3.2. Thermal Stability Measurements

A commercial TGA/DTA (Bähr STA 503) was used to perform the isothermal TG experiments. He (99.998%) was used as carrier gas in DTA/TG experiments. The flow rate of 100 cm³/min was controlled by a calibrated mass flow controller. This flow rate was found to be sufficient to ensure that the concentration of substance at the top of the crucible remains nearly zero throughout the measurement as proved experimentally: a change in flow rate did not measurably change the mass loss rate. Open alumina crucibles were used throughout the experiments with an inner diameter of 5.3 mm and inner depth of 7.2 mm. Exactly-weighed samples from 5 to 20 mg were used in TG experiments. The temperature sensor was calibrated by measuring the melting points of reference substances (4-nitrotoluene, naphthalene, indium and potassium perchlorate), which cover the whole temperature range for the measurements. The pressure was atmospheric throughout.

### 3.3. Vapor Pressure Measurements

**Figure 6:**
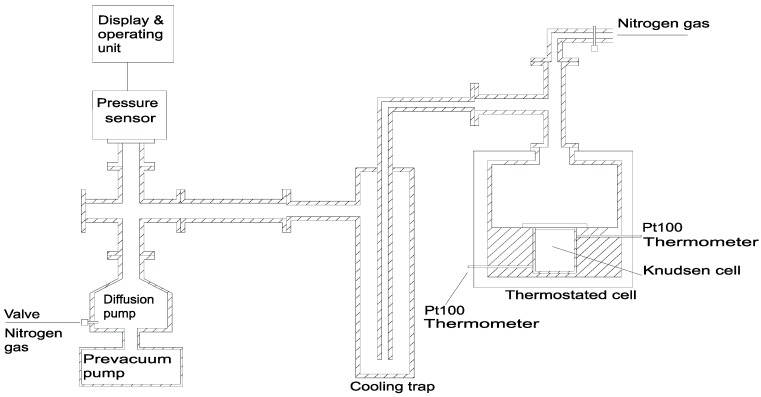
Experimental setup for measuring the vapor pressure.

A home-built stainless steel Knudsen cell was used for vapor pressure measurements. The experimental setup is described in previous publications [18,23] and hence, only a brief description is given here. The schematic diagram is shown in Figure 6. 

The setup includes a Knudsen cell, two Pt100 thermometers, a stainless steel thermostated vessel (vacuum chamber), a cooling trap, a diffusion pump, a pre-vacuum pump, a pressure sensor with a display and an operating unit and arrangement for flushing inert gas (e.g. nitrogen). The Knudsen cell is situated in a vacuum chamber, with good thermal contact around the cell. The temperature of the stainless steel chamber is controlled with a PID temperature controller. The heating was done with an electrical band heater which was wrapped around the chamber carefully to cover it completely. The outer side was then covered with insulation material. The temperature was measured at two different places inside the chamber and did not differ more than 0.1 K. The difference between the actual evaporation temperature (inside the Knudsen cell) and the measured chamber temperature was determined in many experiments performed before the actual measurements by bringing a calibrated Pt-100 thermometer inside the Knudsen cell and measuring its temperature. This temperature difference which was different for different temperatures was always taken into account to correct the display temperature. It was ensured that the thermal equilibrium between the sample and the thermal reservoir (chamber) was attained. A provision for circulating the nitrogen gas was made in order to prevent the degradation of substances by atmospheric air and moisture before the evacuation. This was done by making an inlet at the top of heating cell for introducing the nitrogen into experimental setup and an outlet through a valve in the diffusion pump. In this way the circulation of nitrogen was ensured during the heating period.

A well defined amount of the substance (50–100 mg), depending upon the temperature of the measurement and the substance, was weighed (accuracy: 0.03 mg) into the cell. The filling and weighing was done under inert gas atmosphere in a glove box. The cell was then tightened and put into the vacuum chamber. The thickness of the aluminum foil used was 70 μm. The temperature of the chamber was maintained constant to better than ± 0.2 °C. Prior to evacuation, enough time (at least 60 minutes) was allowed for the attainment of a constant temperature, which was recorded with the help of a calibrated Pt-100 thermometer. During this time nitrogen atmosphere was maintained in the chamber. It was ensured that the substance evaporated before evacuating the cell was less than the accuracy of measurement. The evacuation of the chamber was then started and the time interval was measured between the time when the vacuum reached the pressure of around 10^-3^ Pa and the time when the high vacuum pump was turned off and the pressure was above 10^-3^ Pa. Typical times were 1−10 hours (in this time the weight losses were between 3 and 50 mg depending on the hole size (ranging from 0.5 to 0.9mm), the temperature and the substance). The cell was then brought to room temperature in a desiccator and weighed again. The system was regularly tested with reference substances (ferrocene, phenanthrene and anthracene) having different vapor pressures, and proved to furnish reliable results over a large temperature range. More details are given in previous papers [18,23]. The uncertainties in the evaporation time and in the mass loss are estimated to be 0.5 minutes and 0.05 mg, respectively. In the evaluation of the data, no additional calibration was performed. The maximum overall uncertainty in vapor pressure measurements was estimated to be ± 0.1 to ± 1.0 Pa in the pressure range of 10-50 Pa and ± 0.02 to ± 0.2 Pa in the pressure range of 0.4−10 Pa. This overall uncertainty was calculated assuming that the uncertainties in evaporation time, mass loss and the correction factor are independent and random. 

## 4. Conclusions

The vapor pressures of ruthenocenes **1**−**11** (Figure 1) were investigated. It was found that **5**, **7** and **8** start to decompose at typical evaporator conditions at the isothermal temperature of 95 °C (**5**) and 143 °C (**7** and **8**). As for thermally unstable compounds, correct vapor pressure measurements are not possible since the mass loss may be due to evaporation as well as due to reactions to volatile products. All the gravimetric approaches to measure the vapor pressure of thermally unstable precursors will fail as soon as the decomposition plays a role, because decomposition products will evaporate along with the precursor molecules. Due to this, a measurement of the vapor pressure of these compounds was not possible. Whereas, the molecules **1**−**4**, **6** and **9**−**11** are thermally stable in the measured temperature range, hence their sublimation pressures were measured at various temperatures. 

[(*η*^5^‑C_5_H_4_SiMe_3_)_2_Ru] (**11**) was found to be highly volatile, notwithstanding its high molecular weight. It melts at a temperature of 55 °C. The vapor pressure of **11** varied from 2.2 Pa at 59 °C to 6.3 Pa at 68 °C. The sublimation pressures of [(*η*^5^‑C_5_H_5_)_2_Ru] (**1**), [(*η*^5^‑C_5_H_5_)-(*η*^5^‑C_5_H_4_CH_2_NMe_2_)Ru] (**3**) and [(*η*^5^‑C_7_H_11_)_2_Ru] (**4**) are of the same order of magnitude, just a little lower than the one for [(*η*^5^‑C_5_H_4_SiMe_3_)_2_Ru] (**11**). The asymmetrically substituted sandwich molecule [(*η*^5^‑C_5_H_5_)--(*η*^5^‑C_5_H_4_CH_2_NMe_2_)Ru] (**3**) is also easily to sublime. For the ‘open metallocene’ [(*η*^5^‑C_7_H_11_)_2_Ru] (**4**) a literature value is given [16]. It agrees very well with the data we measured using the Knudsen effusion method. In comparison with the analog [(*η*^5^‑OC_6_H_9_)_2_Ru] (**2**), the vapor pressure of [(*η*^5^‑C_7_H_11_)_2_Ru] (**4**) is much higher, due to the absence of the oxygen atoms. Ruthenocene **6** was found to have the lowest sublimation pressure although it has a low molecular mass. For **11**, **3**, **1**, **4**, **2**, **10**, **6** and **9,** a pressure of 1 Pa is reached at 52.2, 53.2, 55, 57, 90, 115, 120, and 125.5 °C, respectively.

Compounds **9** and **10** are asymmetrically substituted by a keto-ester function and show vapor pressures comparable to **6** with two keto groups in the molecular structure. Little differences are noticeable between the results of **9** and **10**. As a consequence of the absence of one CH_2_ group, **10** has an insignificantly higher vapor pressure than **9**. The order of the vapor/sublimation pressure is *p*(**11**) > *p*(**3**) > *p*(**1**) > *p*(**4)** > *p*(**2**) > *p*(**10**) > *p*(**6**) > *p*(**9**). 

The enthalpy of sublimation is determined from the temperature dependence determined from the vapor/sublimation pressure. For the analyzed ruthenocenes, the enthalpy of sublimation follows the order ΔH_sub_(**11**) > ΔH_sub_(**6**) > ΔH_sub_(**10**) > ΔH_sub_(**9**) = ΔH_sub_(**2**) > ΔH_sub_(**1**) = ΔH_sub_(**4**) > ΔH_sub_(**3**). The value for **1** is in a good agreement with the literature data, although a different temperature range was used. Compounds **5**, **7** and **8** were found to be decomposing in isothermal TG experiments (see above) due to the formation of large quantities of a residue. Therefore, they are not recommended to be used as CVD precursors. Optimization of the process parameters and further investigations regarding the decomposition mechanisms were not the central point of the present work, but the analyses of the by-products and impurities are recommended for further studies and can help to improve the quality of the yielded films. The remaining compounds **1–4**, **6**, **9–11** are found to be thermally stable in the investigated temperature range. If coating is to be done at lower temperature, then **11** is recommended as it has higher vapor pressure. Other compounds (**1–4**, **6** and **9–10**) are also suitable to be used as potential CVD precursors. Oxygen containing compounds (**2** and **6**) have relatively low vapor pressure and may be interesting for special purposes.
